# Metrological Evaluation of Metopimazine HPLC Assay: ISO-GUM and Monte Carlo Simulation Approaches

**DOI:** 10.3390/pharmaceutics17101316

**Published:** 2025-10-10

**Authors:** Hasnaa Haidara, Eman A. Assirey, Taoufiq Saffaj, Bouchaib Ihssane

**Affiliations:** 1 Laboratory of Organic Chemistry, Catalysis, and Environment, Faculty of Sciences, Ibn Tofail University, 133, Kenitra 14000, Morocco; 2 Laboratory of Applied Organic Chemistry, Faculty of Sciences and Technology, Fez-Sidi Mohamed Ben Abdellah University, Immouzer Road, Fez 2202, Morocco; taoufiq.saffaj@usmba.ac.ma; 3 Department of Chemistry, College of Science, Taibah University, Al-Madinah Al-Munawarah 41411, Saudi Arabia; eassirey@taibahu.edu.sa; 4 Laboratory of Inorganic and Organic Materials (LPCMIO), Materials Science Center (MSC), School Normal Superior Physio-Chemical, University Mohammed V, ENS Avenue Mohamed Bel Hassan El Ouazzani, Takaddoum, Rabat 5118, Morocco; chouaibihssane@yahoo.fr

**Keywords:** bottom-up approach, measurement uncertainty, HPLC-UV, uncertainty propagation, Monte-Carlo simulation, Metopimazine

## Abstract

**Background**: Measurement uncertainty (MU) is a crucial parameter for ensuring the reliability of analytical methods and the validity of results, as required by ISO 17025:2017. Its estimation is particularly critical for quality control laboratories, where compliance decisions are based on a rigorous interpretation of uncertainties. **Methods**: In this study, we evaluated the uncertainty associated with an HPLC-UV method for the determination of Metopimazine (MPZ) in a pharmaceutical form, applying two complementary approaches: The ISO-GUM (Guide to the Expression of Uncertainty in Measurement) top-down approach and the Monte Carlo Simulation (MCS). **Results**: The results of both approaches showed excellent agreement, thus validating the robustness of the evaluation. The analysis of uncertainty sources revealed that the accuracy of the sample volume (V_Sample_) and the calibration standard (C_x_) were the dominant contributors, representing 39.9% and 36.2% of the total uncertainty, respectively. Combined, these two factors accounted for 76.1% of the variability, underscoring their critical impact on the assay’s precision. The expanded uncertainty (k = 2, 95% confidence level) was determined to be (99.41 ± 0.69)%, reflecting the method’s reproducibility. **Conclusions**: These results highlight the importance of rigorously controlling calibration standard preparation, sample volume, and repeatability conditions to optimize the reliability of the assay.

## 1. Introduction

**Figure 1:**
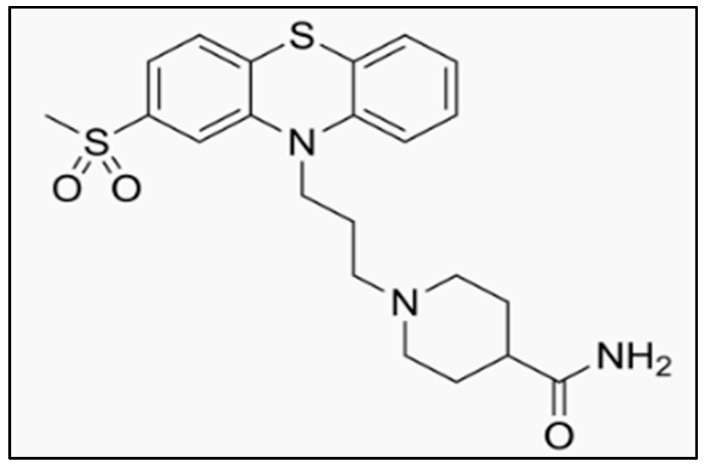
Structure of 1-[3-(2-methylsulfonylphenothiazin-10-yl)propyl]piperidine-4-carboxamide (Metopimazine).

Metopimazine (MPZ), an antiemetic agent belonging to the phenothiazine class, is clinically used to treat nausea and vomiting, including those associated with cancer chemotherapy. Its chemical structure, defined as 1-[3-[2-(methylsulfonyl)-10H-phenothiazin-10-yl]propyl]-4-piperidinecarboxamide (Figure 1), acts by blocking central dopamine receptors, thereby alleviating emetic symptoms [1]. For its quantification in pharmaceutical formulations, high-performance liquid chromatography (HPLC) coupled with UV detection remains the gold-standard method, offering efficient separation, high selectivity, and sensitivity suitable for accurately quantifying MPZ, even at low concentrations [2,3].

In the pharmaceutical industry, where drug quality and safety are paramount, the evaluation of measurement uncertainty (MU) is critical to ensure the reliability of analytical results [4,5]. MU quantifies the inherent variability in measurements, defining a confidence range around results, which is vital for regulatory compliance and patient safety [6,7,8]. Specifically, demonstrating competence in MU estimation is a mandatory requirement for laboratories accredited under ISO/IEC 17025 [9], and is increasingly expected by pharmacopeias (e.g., USP 1010) to support conformity assessments, the critical decision of whether a batch meets its quality specification. Although often overlooked, its importance is emphasized by organizations such as the World Health Organization (WHO) and the European Medicines Agency (EMA), which advocate for its integration into good laboratory practices [9,10,11].

The Guide to the Expression of Uncertainty in Measurement (GUM) provides a standardized framework for MU evaluation by identifying and combining all error sources (bottom-up approach) [7,10,12]. This method, successfully applied in diverse contexts (e.g., metrological surveillance [4], dissolution testing [13], HPLC-UV analyses [14]), remains the benchmark. However, GUM Supplement 1 introduces a complementary approach based on MCS, which is particularly advantageous for complex or nonlinear models. MCS generates probability distributions through numerical iterations, offering greater flexibility and avoiding analytical approximations [15,16,17]. This makes it a promising tool for pharmaceutical analytical methods.

In this study, we evaluate the MU associated with the HPLC-UV quantification of MPZ by applying both the GUM bottom-up approach and MCS. While comparing these methodologies is not new, we used them to Metopimazine for specific reasons that provide distinct novelty. A clear gap in the literature warrants this investigation. Despite the established role of Metopimazine as a first-line antiemetic, a thorough assessment of the measurement uncertainty for its HPLC-UV assay is lacking. This absence represents a regulatory shortfall, especially given the vulnerable patient population it serves. Given MPZ’s critical role as an antiemetic in vulnerable populations, such as those undergoing chemotherapy, the utmost precision in its dosage is paramount. This study provides the first rigorously validated uncertainty framework specifically for this high-stakes compound. Furthermore, the complex mathematical model for MPZ content determination provides an ideal case to test the convergence of both methods on a challenging assay. A key outcome is the identification of the dominant uncertainty sources (sample volume and calibration), offering direct, actionable insights for quality control. Finally, by demonstrating strong agreement between the methods, this work provides a robust justification for laboratories to confidently employ the standardized ISO-GUM approach for this specific assay, ensuring regulatory compliance while leveraging MCS as a powerful validation tool. The objectives are to (1) identify key uncertainty sources (e.g., sample preparation, calibration, repeatability), (2) compare the results obtained from both methods, and (3) discuss their respective advantages within the pharmaceutical regulatory context. This comparative analysis aims to enhance the robustness of analytical methods, aligning laboratory practices with international standards and strengthening confidence in drug quality.

## 2. Materials and Methods

### 2.1. Reagents and Solvents

Reagents and solvents used for chromatographic analysis were of HPLC-grade purity: Milli-Q ultrapure water (double-distilled), Metopimazine reference standard, acetonitrile (ACN), potassium dihydrogen phosphate, and di-potassium hydrogen phosphate. This ensures compatibility with the analytical method and minimizes interference during testing.

### 2.2. Equipment

The HPLC-UV analyses were performed using a Dionex Ultimate 3000 liquid chromatograph (Thermo Fisher Scientific, Waltham, MA, USA), a fully automated system comprising an Ultimate 3000 pump, an autosampler with a 200-position sample tray, a thermostated column compartment, and a UV/VIS detector. The entire system was managed by a computer running Chromeleon software (version 6.8, Thermo Fisher Scientific) for instrument control and data acquisition.

Weighing was performed using a calibrated Mettler analytical balance (Mettler-Toledo, Greifensee, Switzerland). Class A volumetric glassware was used for all sample and standard preparations to ensure measurement accuracy.

### 2.3. Chromatographic Conditions

Mobile phase: The mobile phase consisted of a degassed mixture of 65% phosphate-buffer solution and 35% acetonitrile. The phosphate buffer was prepared by dissolving 2.72 g of potassium dihydrogen phosphate and 3.48 g of dipotassium hydrogen phosphate in purified water within a 1 L volumetric flask. The solution was then diluted to the mark with purified water to achieve a final volume of 1000 mL. The pH of the resulting buffer solution was approximately 6.8, as determined by the equilibrium of the potassium dihydrogen phosphate and dipotassium hydrogen phosphate salts.

Stationary phase: A C8 column (15 cm × 3.9 mm internal diameter, 5 µm particle size; e.g., Waters XBridge C8, Waters, Milford, MA, USA).

Column temperature: Maintained at a controlled laboratory temperature of 22 ± 2 °C. Mobile phase flow rate: 1 mL/min. Detection wavelength: 240 nm. Injection volume: 10 µL.

### 2.4. Method Validation

System Suitability Testing (SST): Before commencing the analytical procedure, system suitability was verified to ensure the chromatographic system’s performance was adequate. The test was performed by injecting six replicates of 10 µL of the standard solution. The pre-defined acceptance criteria, established during method validation, were as follows:

- Retention time of Metopimazine: approximately 4 min.
- Tailing factor: ≤1.2.
- Number of theoretical plates: ≥2500.
- The percentage coefficient of variation (% CV) of the peak areas from the six replicate injections must be less than 2.0%.
- The % CV of the retention times from the six replicate injections must be less than 1.0%.

These criteria, which align with the requirements of pharmacopeial standards such as USP <621> and Ph. Eur. 2.2.46, ensure the precision of the injection system, the stability of the chromatographic conditions, and the quality of the peak shape for accurate integration.

The system performance was found to meet all acceptance criteria, thereby validating the subsequent uncertainty calculations.

The HPLC-UV method used for the quantification of Metopimazine was fully validated in accordance with the ICH Q2 (R1) guideline for its intended purpose as an assay for the active pharmaceutical ingredient. The key validation parameters were assessed as follows:

Specificity: The method demonstrated excellent specificity, with no interference observed from excipients or placebo at the retention time of Metopimazine, confirming its specificity for the analyte.

Accuracy: The accuracy was evaluated by recovery studies using a standard addition technique. The mean recovery was 100.32% (for *n* = 12 samples) with a percentage relative standard deviation (% RSD) of less than 0.74%. The 95% confidence interval for the mean recovery (99.85–100.79%) included 100%, confirming the method’s accuracy.

Precision: The precision was verified through repeatability (intra-day) and intermediate precision (inter-day) studies. The % RSD for both repeatability (0.77%) and intermediate precision (0.88%) was less than 2.0%, confirming the excellent precision of the method.

Linearity and Range: The method showed excellent linearity over a concentration range of 0.12 to 0.28 mg/mL (60% to 140% of the target concentration). The correlation coefficient (*R*^2^) was ≥0.999. Statistical tests (F-test, *t*-test) confirmed a significant slope.

Robustness: The method was found to be robust for deliberate variations in key parameters such as mobile phase composition, flow rate, and column temperature. The system suitability criteria were met in all instances during testing.

All results confirmed that the method is specific, accurate, precise, linear, and robust for the reliable quantification of Metopimazine in its injectable form and provides a solid foundation for the measurement uncertainty study.

## 3. Results

### 3.1. Uncertainty Assessment via the GUM Methodology

The Guide to the Expression of Uncertainty in Measurement (GUM), standardized as ISO/IEC Guide 98-3:2008 [18], provides internationally recognized guidelines for evaluating and reporting measurement uncertainty. Practical applications of GUM in analytical chemistry [11], such as the Eurachem/CITAC guide Quantifying Uncertainty in Analytical Measurement, illustrate its adaptation to chemical analyses [10].

The GUM method employs a bottom-up methodology to estimate total measurement uncertainty by systematically identifying, quantifying, and combining all uncertainty sources. This process relies on defining a measurement equation:

*Y* = *f*(*X*_1_, *X*_2_…, *X*_*n*_) (1)

where *Y* represents the measurand (analytical result) and *X_i_* denotes input parameters influencing *Y.*

An estimated value *y* for *Y* is derived by substituting input estimates *x*_1_, *x_2_*, …, *x_n_ x*_1_, *x*_2_, *…*, *x_n_* into the equation:

*y* = *f*(*x*_1_, *x*_2_, …, *x*_*n*_) (2)

The combined standard uncertainty $u\left(y\right)$ is calculated by propagating the uncertainties of the input estimates $u(x_{i})$ using the law of uncertainty propagation. For uncorrelated inputs, this is expressed as:

(3) $$u^{2}\left(y\right)=\sum_{i=1}^{n}\left({c_{i}}^{2}u^{2}\left(x_{i}\right)\right)$$

where $C_{i}$ represents sensitivity coefficients (partial derivatives $\frac{\;\partial f}{{\partial x}_{i}}$) that quantify how y changes with variations in *x_i_*; each uncertainty contribution is calculated as:

(4) $$u_{i}\left(y\right)=c_{i}.u(x_{i})$$

Standard uncertainties $u\left(x_{i}\right)$ can be assessed using non-statistical means (Type B) or by observing repeated experiments (Type A evaluation).

In type A evaluation, the standard uncertainty $u\left(x_{i}\right)$ is the standard deviation of the mean of m measurements:

(5) $$u\left(x_{i}\right)=S(\bar{x_{i}})=\frac{S(x_{i})}{\sqrt{m}}$$

where $S(x_{i})$ is the sample standard deviation, and degrees of freedom $\nu_{i}=m-1.$

The expanded uncertainty (U) establishes a range around the measurement result where the true value of the measurand is expected to lie with a specified level of confidence. It is calculated by scaling the combined standard uncertainty *u*(*y*) by a coverage factor (k), which reflects the desired confidence level:

(6) U=k.u(y)

For a normally distributed measurand, a coverage factor of k = 2 corresponds to approximately 95% confidence, while k = 3 extends the confidence interval to roughly 99.7%. This scaling ensures that the reported uncertainty aligns with the measurement’s intended statistical reliability.

However, it is sometimes recommended to select the value of *k* based on the two-tailed Student’s t associated with the degrees of freedom of the contribution, considering the required confidence level (often 95%) and the effective degrees of freedom, $\nu_{eff}$, calculated using the Welch–Satterthwaite formula [19,20].

(7) νeff=u4(y)∑iui4(y)νi

where the degrees of freedom of $u(x_{i})$ are represented by $\nu_{i}$. The extended uncertainty $U_{p}$ that results from the value of k, which is commonly represented as $k_{p\;}$, and where p is the confidence probability, will keep the coverage probability at about the necessary level p (typically 95%).

### 3.2. Uncertainty Assessment via Monte Carlo Method

The MCS method, as outlined in GUM Supplement 1 [21], provides a numerical approach for estimating measurement uncertainty by propagating probability distributions through a computational model. Unlike the classical GUM framework, which relies on linear approximations, MCS employs random sampling to simulate thousands (or millions) of possible measurement scenarios, providing a more robust solution for complex or nonlinear models [22].

In this approach, each input quantity $X_{i}$ is assigned to a probability distribution (e.g., normal, rectangular, or triangular) based on available knowledge. MATLAB 2020b is particularly well-suited for implementing MCS due to its powerful statistical and computational capabilities. A MATLAB script can be developed to: (1) define the measurement function *y* = *f*(*x*_1_, *x*_2_, …., *x_n_*), (2) generate random samples from the input distributions, (3) evaluate the model for each sample set, and (4) analyze the resulting distribution of y to determine the output uncertainty.

To ensure full reproducibility, the MATLAB random number generator was initialized with a fixed seed. A convergence study was performed by simulating an increasing number of trials (*M* = 10^4^, 10^5^, and 10^6^). The 95% coverage interval width stabilized within 0.01% for *M* > 10^5^, confirming that *M* = 10^6^ was sufficient for a precise and stable outcome. Since no significant correlations were identified between the input uncertainty sources during method development, all quantities were sampled independently in the simulation.

Key metrics such as the mean, standard deviation (standard uncertainty), and 95% coverage interval are directly derived from the simulated output distribution.

This method eliminates the need for sensitivity coefficients and provides a more intuitive representation of uncertainty, especially when dealing with asymmetric or non-Gaussian distributions [23,24]. MATLAB’s built-in functions (e.g., randn, random, and histogram tools) streamline the implementation, making MCS accessible for advanced uncertainty analysis in metrology and analytical chemistry.

### 3.3. Validation of GUM Results Using MCS

The MCS method serves as a robust validation tool for uncertainty estimates derived from the GUM approach. This validation process, as outlined in GUM Supplement 1, involves comparing the expanded confidence intervals generated by both methods to assess their agreement. The procedure requires that the intervals align within a predefined numerical tolerance, defined as:

(8) $$\delta =0.5\times \;10^{\;l}$$

where l is an integer, and $u\left(y\right)=c.10^{\;l}$ represents the standard uncertainty from the GUM method, with *c* being an integer of 1 or 2 significant digits (depending on validation precision requirements).

#### 3.3.1. GUM-Derived Interval

The lower and upper bounds (*L_low_* and *L_high_*) are calculated as:

(9) $$L_{low}=y-U_{P\;\%},\;L_{high}=y+U_{P\;\%}$$

where $U_{P\;\%}=k.u\left(y\right)$ is the expanded uncertainty for a confidence level *P* %, and k is the coverage factor corresponding to *P* %.

#### 3.3.2. MCS-Derived Interval

The bounds $y_{low}$ and $y_{high}$ are extracted directly from the MCS distribution of the measurand [25].

#### 3.3.3. Decision Criteria

The absolute differences between the intervals are computed as:

(10) $$d_{low}=\left|L_{low}-y_{low}\right|\text{ \& }d_{high}=\left|L_{high}-y_{high}\right|$$

If both ${\;d}_{low}$ and $d_{high}$ are less than *δ*, the GUM results are validated, confirming the applicability of its assumptions. If not, the MCS method or alternative approaches (e.g., the total error method [26]) should be adopted for more reliable uncertainty estimation.

This comparative analysis ensures the robustness of uncertainty quantification, particularly when the GUM’s linearity assumptions may not hold. The MCS validation thus acts as a critical checkpoint for methodological rigor in metrology and analytical chemistry [27].

### 3.4. Uncertainty Evaluation in Analytical Measurements: A GUM-Based Approach

#### 3.4.1. Definition of the Measurand

The initial phase involves a comprehensive characterization of the measurement process. This requires: (1) systematic documentation of all analytical steps in the measurement procedure, and (2) development of a mathematical model that precisely defines the measurand and its functional relationship with all influencing parameters. This foundational step establishes the framework for subsequent uncertainty quantification in the analytical method.

**Figure 2:**
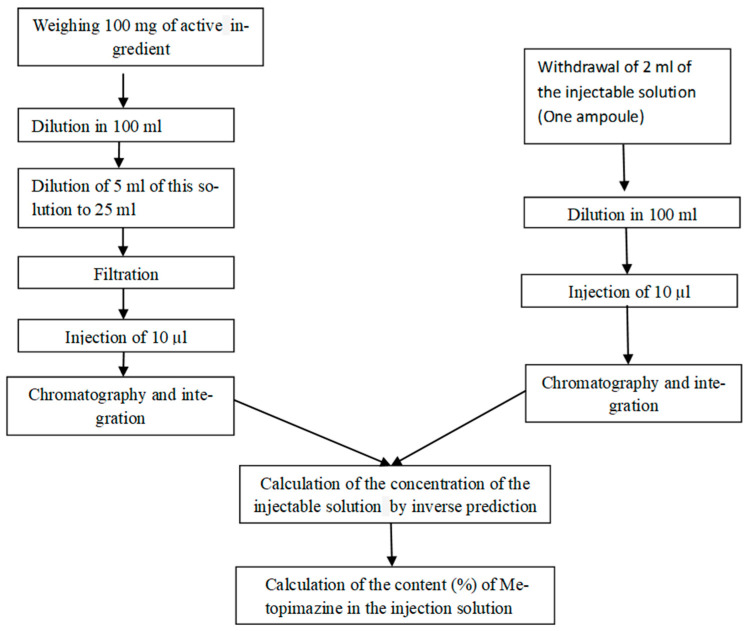
Flowchart of the analytical procedure for the assay of Metopimazine by HPLC-UV analysis.

The quantification of Metopimazine content (%) is performed using the following analytical procedure (Figure 2).

The measurand is the content of Metopimazine expressed in (%) in an ampoule of the injectable solution, which depends on the mass of the active principle, its purity, the analytical response, as well as the surface area of the solution to be examined, and the volume dilution of these two solutions:

(11) $$C\left(\%\right)=\left(\frac{A_{t}}{A_{st}}\right)\times \left(\frac{P_{t}}{V_{Sample}}\right)\times \left(\frac{{df}_{st}}{{df}_{t}}\right)\times \left(\frac{P}{100}\right)\times \left(\frac{V_{m}}{Dose}\right)\times 100$$

$A_{t}$: Area of the peak of Metopimazine in the test solution.

$A_{st}$: Peak area of Metopimazine in the standard solution

$P_{t}$: weight of reference substance in mg.

$V_{Sample}$: Volume of specimen in mL.

${fd}_{st}$: Dilution Factor of the standard solution in mL^−1^.

${fd}_{t}$: dilution Factor of the solution to be examined in mL^−1^

P: Purity of the reference Metopimazine in %.

$\frac{V_{m}}{Dose\;}$: Ratio between the average volume of the injectable solution and the corresponding quantity of Metopimazine. This ratio is 1 mL/10 mg.

With: ${fd}_{st}=\frac{1}{{Vt}_{1}}*\frac{V_{p}}{{Vt}_{2}}{;\;fd}_{t}=\frac{1}{V_{t}}$

So:

(12) $$C\left(\%\right)=\left(\frac{A_{t}}{A_{st}}\right)\times \left(\frac{P_{t}}{{Vt}_{1}}\right)\times \left(\frac{V_{p}}{{Vt}_{2}}\right)\times \left(\frac{V_{t}}{V_{Sample}}\right)\times \left(\frac{P}{100}\right)\times \left(\frac{V_{m}}{Dose}\right)\times 100$$

${Vt}_{1}$: Dilution volume of standard stock solution.

Vp: Volume taken from the standard stock solution.

${Vt}_{2}$: Dilution volume of volume taken from the stock solution.

$V_{t}$: Volume of the test solution.

#### 3.4.2. Identification and Analysis of Sources of Uncertainty

**Figure 3:**
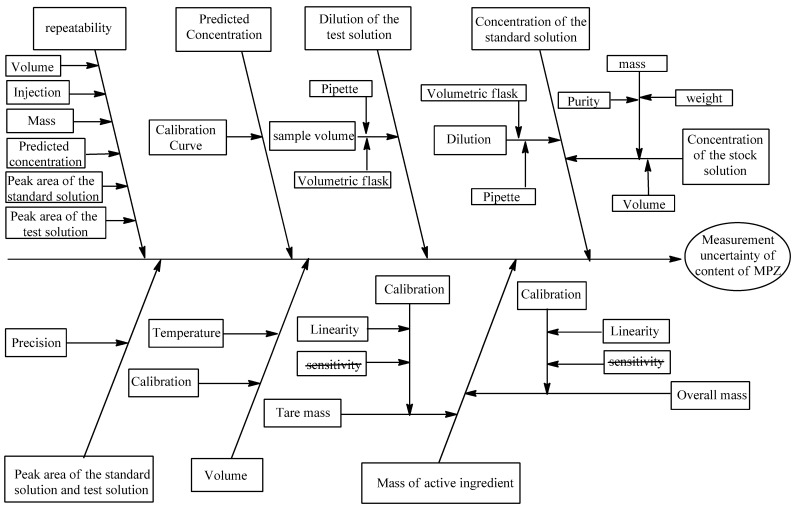
Ishikawa diagram of uncertainty sources for HPLC-UV analysis.

This section systematically examines potential contributors to measurement variability in the analytical procedure. It focuses on developing an Ishikawa diagram (Figure 3) to systematically identify and categorize all potential sources of uncertainty in the measurement process.

When weighing is performed using the same balance within a narrow mass range, the sensitivity contribution may be considered negligible, simplifying the uncertainty analysis.

##### Purity Specification

The certified reference standard of Metopimazine has a documented purity of 99.99 ± 0.01% (certificate value), which is therefore equal to 0.9999 ± 0.0001.

##### Mass Measurement

The mass measurement step involves weighing 100 mg of active ingredient using a tared weighing method to prepare the test solution. According to the balance manufacturer’s specifications, this measurement is subject to three main sources of uncertainty: weighing repeatability, the digital resolution of the balance display, and uncertainty in the balance calibration function. The calibration uncertainty itself includes contributions from both the balance sensitivity and its linear characteristics. This comprehensive accounting of measurement variability ensures proper characterization of the mass uncertainty in the analytical procedure.

##### Volume

The measurement of solution volume using pipettes and volumetric flasks involves three primary uncertainty components:

- Glassware calibration tolerance: Uncertainty in the certified volume of the volumetric flask and pipette.
- Filling variability: Variation when adjusting the meniscus to the calibration mark.
- Temperature deviation: Difference between the working temperature and the glassware’s calibration temperature (typically 20 °C).

##### Peak Area Uncertainty

The quantification of Metopimazine concentration depends directly on integrated peak areas (*A_t_*, *A_st_*), which are subject to various sources of chromatographic uncertainty. As highlighted in the review by Vicki and Barwick, key factors affecting peak area variability include mobile phase composition, flow rate stability, column temperature control, UV detection parameters, and injection system performance. These parameters influence both retention time reproducibility and peak integration accuracy. The impact of these variables was evaluated through system suitability testing by analyzing the precision of retention times and peak areas.

##### Predicted Concentration

Metopimazine concentration is determined by applying its measured response to the established calibration curve equation before final quantification.

(13) $$A_{t}=a_{0}+a_{1}.c_{x}$$

(14) $$C_{x}=\frac{\left(A_{t}-a_{0}\right)}{a_{1}}$$

(15) $$u\left(C_{x}\right)=s=\frac{S_{res}}{a_{1}}\times \sqrt{\frac{1}{p}+\frac{1}{n}+\frac{{\left(C_{x}-\bar{C}\right)}^{2}}{{SSD}_{x}}}$$

With:

$a_{1}$: Slope of the calibration curve

p: Number of measurements to determine $C_{x}$.

*n*: Number of measurements necessary for calibration

$C_{x}$: Predicted concentration

$\bar{C}$: Average value of the concentration of different standards for calibration

$a_{0}$: intercept of the calibration curve

${SSD}_{x}$: Sum of squares of the deviations of the concentration variable.

#### 3.4.3. Qualification of Uncertainty Components

The third step involves quantifying the variability of each uncertainty source identified in the previous stage and expressing it as standard uncertainties. For empirical data obtained through repeated measurements, Type A evaluation is applied by calculating the standard deviation of the mean. Type B evaluation is employed using manufacturer specifications, calibration certificates, or scientific literature for documented or theoretical uncertainties. All uncertainty contributions must be converted to standard uncertainties at a 1σ confidence level to ensure consistent units before combination. This standardization process enables proper propagation of uncertainties through the measurement model.

##### Evaluation of Precision Uncertainty

The method’s precision was assessed by executing the complete analytical procedure according to the established operating protocol. Rather than evaluating individual repeatability contributions from each potential source, all variability components are collectively represented through a comprehensive study of repeatability.

This assessment involved conducting three independent analytical runs, each comprising six replicate determinations. The combined repeatability value, derived from these 18 total measurements, was calculated as follows:

(16) $$S_{R}=\frac{S_{r}}{\sqrt{n}}$$

The method precision evaluation yielded a standard deviation of 0.0005 and relative uncertainty $S_{R}$ = 0.0005.

This sample size (*n* = 18) exceeds the minimum recommendations for obtaining a reliable estimate of the standard deviation in uncertainty budgets, as outlined in metrological guides such as EURACHEM. With 17 degrees of freedom, the relative standard error of the standard deviation estimate is approximately 17%, which is considered adequate for reliable precision estimation in pharmaceutical quality control.

##### Purity Uncertainty

The certificate of analysis for Metopimazine specifies a purity of 0.9999 ± 0.0001. Without information about the distribution type, a rectangular distribution is conservatively assumed. The standard uncertainty $u_{P}$ is therefore calculated by dividing the stated uncertainty by $\sqrt{3}$.

$$u_{P}=\frac{0.0001}{\sqrt{3}}=0.000058$$

##### Mass Measurement Uncertainty

The uncertainty in the active ingredient mass measurement was determined to be ±0.02 mg based on the balance calibration certificate data, which accounts for all three key uncertainty components: balance repeatability, digital resolution, and calibration function performance (including sensitivity and linearity effects). This comprehensive evaluation ensures proper characterization of the weighing step’s contribution to overall measurement uncertainty.

The uncertainty contribution from the balance linearity is calculated as:

$$u_{lin}=\frac{0.02}{\sqrt{3}}=0.0115\;mg$$

##### Predicted Concentration

The standard uncertainty of the predicted concentration, $u\left(C_{x}\right)$, was a significant contributor to the overall uncertainty budget. Its calculation is based on the regression statistics of the calibration curve, which was rigorously validated according to ICH Q2 (R2) guidelines.

The key statistical parameters obtained from the regression analysis are summarized in Table S1.

Residual analysis was performed to validate the key assumptions of homoscedasticity and normality underlying the uncertainty calculation in Equation (15). The plot of residuals versus predicted concentrations (Supplementary Figure S1) displays a random scatter of points without any discernible systematic pattern, confirming that the variance of the residuals is constant across the concentration range (homoscedasticity). This justifies the use of an unweighted regression model. The normal Q-Q plot (Supplementary Figure S2) shows the majority of residuals closely following the theoretical straight line. The satisfactory alignment, particularly in the central region, supports the assumption of normally distributed residuals. Together, these findings validate the use of the unweighted linear regression model and the associated standard uncertainty formula for *u*(*C_x_*).

The calibration design, with 15 data points across five concentration levels, provides a statistically robust foundation for the regression analysis, ensuring the reliability of the uncertainty estimate *u*(*C_x_*).

The standard uncertainty for the sample concentration prediction was calculated using the formula [15]:

$$u\left(C_{x}\right)=s=\frac{S_{res}}{a_{1}}\times \sqrt{\frac{1}{p}+\frac{1}{n}+\frac{{\left(C_{x}-\bar{C}\right)}^{2}}{{SSD}_{x}}}$$

where *p* = 6 is the number of sample replicates, *n* = 15 is the total number of calibration data points, $C_{x}$ = 0.2005 mg/mL is the predicted sample concentration, and the sum of squares of the deviations of the calibration concentration levels ${\;SSD}_{x}$ is 0.048 (mg/mL)^2^.

The standard uncertainty $u\left(C_{x}\right)$ was calculated as follows:

$$u\left(C_{x}\right)=s=\frac{S_{res}}{a_{1}}\times \sqrt{\frac{1}{p}+\frac{1}{n}+\frac{{\left(C_{x}-\bar{C}\right)}^{2}}{{SSD}_{x}}}$$

$$u\left(C_{x}\right)=s=\frac{0.27}{332.14}\times \sqrt{\frac{1}{6}+\frac{1}{15}+\frac{{\left(0.2005-0.2\right)}^{2}}{0.048}}$$

$$u\left(C_{x}\right)=0.000393\approx 0.0004\;mg/mL$$

This calculated value of 0.0004 mg/mL has been used in the uncertainty budget.

##### Volume Uncertainty

Three primary factors contribute to volume measurement uncertainty: calibration tolerance, repeatability, and temperature effects. The manufacturer’s specifications for glassware calibration at 20 °C indicate the following tolerances:

- 100 mL volumetric flask: ±0.1 mL
- 25 mL volumetric flask: ±0.04 mL
- 5 mL volumetric pipette: ±0.015 mL
- 2 mL volumetric pipette: ±0.01 mL

The selection of probability distributions follows the recommendations in the Eurachem/CITAC Guide QUAM. For volumetric tolerances (calibration uncertainty), a triangular distribution was applied as it represents the more realistic case where values near the nominal specification are most probable due to manufacturing quality control. For temperature effects, a rectangular distribution was used as there is an equal probability of the laboratory temperature being anywhere within the documented ±4 °C variation range. This approach aligns with QUAM on selecting appropriate distributions for different uncertainty components.

The standard calibration uncertainty is therefore derived from the manufacturer’s specified instrument tolerances, modeled using a triangular distribution.

$$u\left(V_{100}\right)=(\frac{0.1}{\sqrt{6}})=0.04082\;mL;\;u\left(V_{25}\right)=(\frac{0.04}{\sqrt{6}})=0.01633\;mL\;;$$

$$u\left(V_{5}\right)=\left(\frac{0.015}{\sqrt{6}}\right)=0.00612\;mL;\;u\left(V_{2}\right)=\left(\frac{0.01}{\sqrt{6}}\right)=0.004082\;mL;$$

- Repeatability

The validation data from method development incorporates all relevant repeatability contributions, including both volume dispensing and weighing operations. This comprehensive approach enables the combined use of these established repeatability values in the current uncertainty budget, ensuring that all procedural variations are properly accounted for.

- Temperature effects

Significant temperature-related uncertainty arises from the discrepancy between the manufacturer’s calibration conditions (20 °C) and actual laboratory conditions (±4 °C variation). For aqueous solutions, the dominant effect comes from liquid expansion rather than glassware expansion. Using water’s volume expansion coefficient (2.1 × 10^−4^ °C^−1^), we calculate temperature-induced uncertainty for each volumetric instrument:100 mL flask, 25 mL flask, 5 mL pipette, and 2 mL pipette.

The calculation formulas for each instrument’s temperature-dependent volume variation will be presented in the following section.

$$\left(100\times 4\times 2.1\times 10^{-4}\right)=0.084\;mL$$

$$\left(25\times 4\times 2.1\times 10^{-4}\right)=0.021\;mL$$

$$\left(5\times 4\times 2.1\times 10^{-4}\right)=0.0042\;mL$$

$$\left(2\times 4\times 2.1\times 10^{-4}\right)=0.00168\;mL$$

We calculate the standard uncertainty assuming that the distribution for the temperature variation is rectangular, i.e.,

$$\;\frac{0.084}{\sqrt{3}}=0.04850;\;\frac{0.021}{\sqrt{3}}=0.01212;\;\frac{0.0042\;}{\sqrt{3}}=0.00242;\;\frac{0.0017\;}{\sqrt{3}}=0.00097$$

The three contributions are combined to give the standard uncertainty *u*(*V*) of each measuring instrument’s volume *V*, as listed in Table 1.

##### Peak Area Uncertainty

System suitability was verified through six replicate injections of the Metopimazine standard solution. The results, summarized in Table 2, confirmed that the chromatographic system performance met all pre-defined acceptance criteria (e.g., % CV for peak area and retention time). The precision data from this test were subsequently used to quantify the standard uncertainty associated with the chromatographic measurement variability (*u*(*HPLC-UV*)). This systematic approach ensures that the uncertainty component related to peak integration is characterized from data that first demonstrated the system’s fitness for purpose.

**Figure 4:**
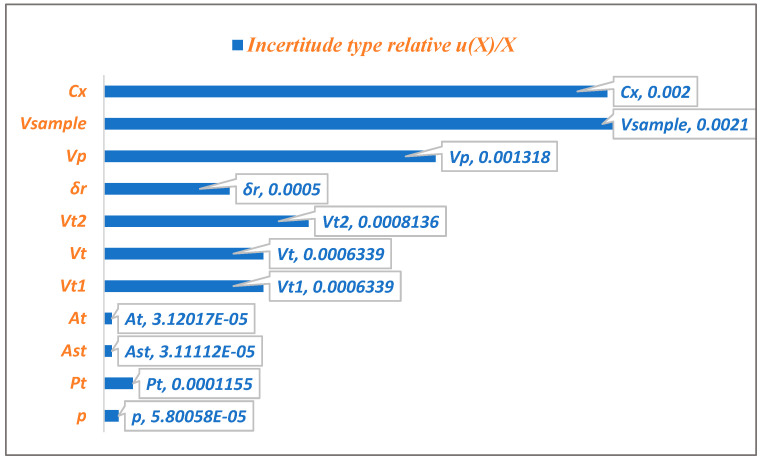
Quantitative Assessment of Uncertainty Contributions in MPZ Content Determination.

Figure 4 illustrates the relative contributions of various uncertainty sources to the combined standard uncertainty of (MPZ) content. The analysis reveals that the predicted concentration contributes most significantly to the overall uncertainty, with the specimen’s volume in mL being the second major contributor.

The uncertainties associated with the dilution volume of the standard stock solution, the dilution volume of the volume taken from the stock solution, and the Volume of the test solution are of comparable magnitude. In contrast, the purity uncertainty, the Area of the peak of Metopimazine in the test solution, and the Peak area of Metopimazine in the standard solution have a negligible impact on total uncertainty.

The Pareto chart (Figure 4) reveals that the specimen volume (*V_Sample_*) and the predicted concentration from the calibration (*Cx*) are the dominant uncertainty sources, collectively responsible for 76.1% of the total variability. Therefore, quality control efforts should prioritize rigorous management of sample pipetting and calibration standard preparation to most effectively reduce overall measurement uncertainty.

#### 3.4.4. Calculation of the Combined Standard Uncertainty

The individual standard uncertainty components identified through Ishikawa analysis are systematically combined to calculate the overall standard uncertainty for the content determination expressed as *C* (%).

After calculating the standard uncertainty associated with each component of the content formula (%) as mentioned in Table 3, we proceed to calculate the compound standard uncertainty.

*C* % is given by (Equation (12)):

$$C\left(\%\right)=\left(\frac{A_{t}}{A_{st}}\;\right)\times \left(\frac{P_{t}}{{Vt}_{1}}\right)\times \left(\frac{V_{p}}{{Vt}_{2}}\right)\times \left(\frac{V_{t}}{P_{w}}\right)\times \left(\frac{P}{100}\right)\times \left(\frac{V_{m}}{Dose}\right)\times 100$$

The estimate of repeatability and the predicted concentration are treated as a relative effect; the complete model equation is therefore:

(17) $$\begin{array}{l} C\left(\%\right)=(\frac{A_{t}}{A_{st}})\times \left(\frac{P_{t}}{{Vt}_{1}}\right)\times \left(\frac{V_{p}}{{Vt}_{2}}\right)\times \left(\frac{V_{t}}{V_{Sample}}\right)\times \left(\frac{P}{100}\right)\times \left(\frac{V_{m}}{Dose}\right)\\ \;\times \delta_{r}\times C_{x}\times 100 \end{array}$$

Using the values grouped in Table 3. The value of the content in % is equal to:

*C* (%) = 99.41%

The uncertainty of repeatability term *u*(*δ_r_*) and the uncertainty from the calibration curve (*u*(*C_x_*)) are considered independent, as the former captures the variability from the entire sample preparation process (weighing, dissolution, dilution), while the latter is specific to the instrumental prediction based on independently prepared calibration standards. The assumption of independence between the repeatability term, *u*(*δr*), and the calibration uncertainty, *u*(*Cx*), is justified by the structure of the measurement model and the origin of the uncertainties, as recommended in the GUM (JCGM 100:2008, 5.1.2) and its Supplement 1 (JCGM 101:2008).

The uncertainties associated with each component are composed according to:

(18) $$\frac{u\left(C\right)}{C}=\sqrt{{\left(\frac{u\left(A^{\prime }t\right)}{A^{\prime }t}\right)}^{2}{+\left(\frac{u\left(A^{\prime }st\right)}{Ast}\right)}^{2}+{\left(\frac{u\left(Pt\right)}{Pt}\right)}^{2}{+\left(\frac{u\left(Vt1\right)}{Vt1}\right)}^{2}+{\left(\frac{u\left(Vp\right)}{Vp}\right)}^{2}{+\left(\frac{u\left(Vt2\right)}{Vt2}\right)}^{2}{+\left(\frac{u\left(Vt\right)}{Vt}\right)}^{2}{+\left(\frac{u\left(V_{Sample}\right)}{V_{Sample}}\right)}^{2}+{\left(\frac{u\left(P\right)}{P}\right)}^{2}+{\left(\frac{u(c_{x})}{c_{x}}\right)}^{2}+{\left(\frac{u(\delta_{rep})}{\delta_{rep}}\right)}^{2}}$$

(19) $$u\left(C\right)=C\sqrt{{\left(\frac{u\left(A^{\prime }t\right)}{A^{\prime }t}\right)}^{2}{+\left(\frac{u\left(A^{\prime }st\right)}{A^{\prime }st}\right)}^{2}+{\left(\frac{u\left(Pt\right)}{Pt}\right)}^{2}{+\left(\frac{u\left(Vt1\right)}{Vt1}\right)}^{2}+{\left(\frac{u\left(Vp\right)}{Vp}\right)}^{2}{+\left(\frac{u\left(Vt2\right)}{Vt2}\right)}^{2}{+\left(\frac{u\left(Vt\right)}{Vt}\right)}^{2}{+\left(\frac{u\left(V_{Sample}\right)}{V_{Sample}}\right)}^{2}+{\left(\frac{u\left(P\right)}{P}\right)}^{2}+{\left(\frac{u\left(c_{x}\right)}{c_{x}}\right)}^{2}+{\left(\frac{u\left(\delta_{rep}\right)}{\delta_{rep}}\right)}^{2}}$$

Using the values grouped in Table 3, the uncertainty of the content (%) of Metopimazine is equal to: $u\left(C\right)$ = 0.3426%

#### 3.4.5. Calculation of Expanded Uncertainty and Result Presentation

The expanded uncertainty *U*(*C*) was calculated by multiplying the combined standard uncertainty by a coverage factor of *k* = 2, corresponding to a confidence level of approximately 95%:

(*C*) = 2 × *u*(*C*) = 0.6852% ≈ 0.69%.

The assay result for Metopimazine in the injectable solution, together with its expanded uncertainty, is therefore: *C* = (99.41 ± 0.69)%.

The acceptability of this result is assessed against the product’s release specification. For the assay of an active pharmaceutical ingredient in a drug product, a common acceptance criterion, aligned with ICH Q6A guidelines, is no less than 95.0% and no more than 105.0% of the label claim (a 10% range). The expanded uncertainty interval of ±0.69% (a 1.38% range) occupies only a small fraction of this specification range. The ratio of the uncertainty interval to the specification range demonstrates that the analytical method is fit-for-purpose, as its uncertainty is sufficiently small to provide a high level of confidence in reliable conformity decisions for pharmaceutical quality control.

### 3.5. Evaluation of Measurement Uncertainty via Simulation Approach

**Figure 5:**
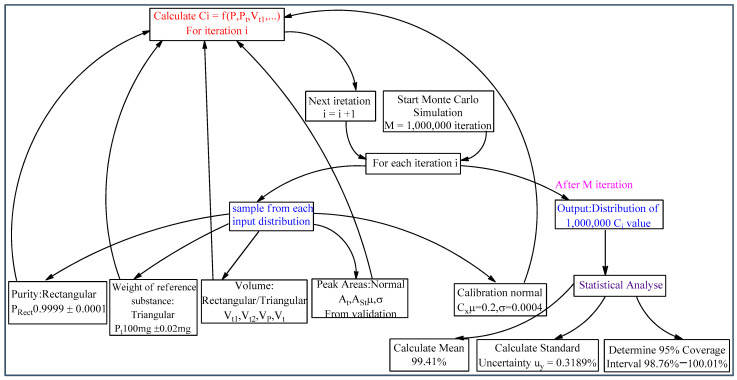
Workflow of the Monte Carlo Simulation.

The GUM analytical approach and MCS numerical method share common steps in uncertainty evaluation, including identifying elementary uncertainty sources and designing measurement models. However, their operating principles differ fundamentally. While GUM uses linearized uncertainty propagation, MCS employs a distribution propagation method to assess combined uncertainty. The MCS approach requires defining probability distribution functions (PDFs) for each input quantity. The selection of probability distributions (Table 4) was based on the origin of the uncertainties and follows recommendations from metrological guidelines such as the GUM and Eurachem/CITAC [9,11]. Figure 5 shows the Workflow of the MCS for uncertainty estimation. The process involves defining probability distributions for all input quantities, running a high number of computational iterations to propagate uncertainty through the measurement model, and analyzing the resulting distribution of the output quantity (MPZ content) to determine the combined uncertainty.

**Figure 6:**
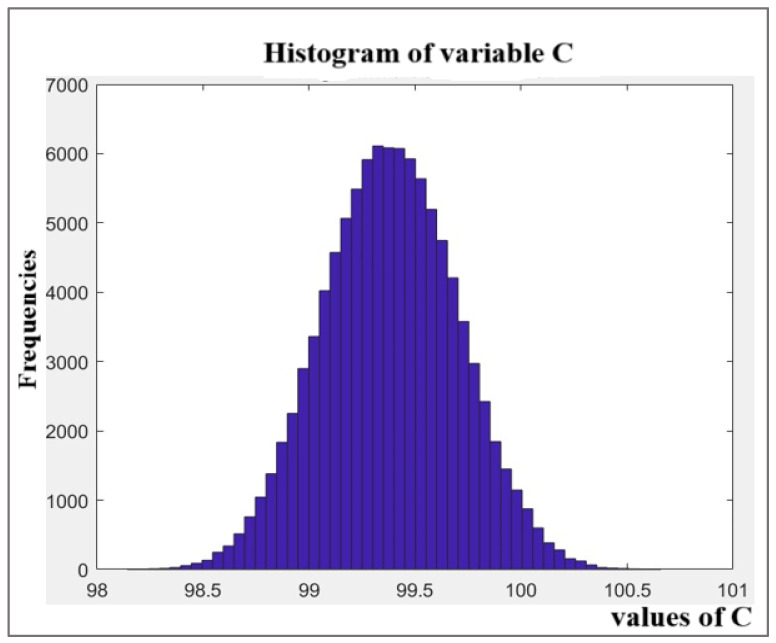
Distribution of the output quantity of the MPZ content.

The number of simulation trials (M) significantly impacts the output quantity’s coverage probability. This study performed M = 10^6^ simulations to ensure the stability of the result. The MCS implementation used MATLAB, with key functions including NORMRND (mean, standard deviation, M, 1) for generating normally distributed data, [*a* + (*b* − *a*) × *RAND*(*M*,1)] for rectangular distributions, and [*a* + (*b* − *a*)/2 × (*RAND*(*M*,1) + *RAND*(*M*,1))] for triangular distributions. Here, *RAND*(*M*,1) generates random numbers between 0 and 1, while a and b represent distribution limits. This methodology effectively propagates input uncertainties through the measurement model, yielding reliable output uncertainty estimates. The output distribution for MPZ content, derived from the MCS, is shown in Figure 6. The distribution is effectively symmetric and approximately normal, which justifies the use of a symmetric coverage interval. It reflects the precise shape of the output distribution and the large number of effective degrees of freedom resulting from combining the input uncertainties. The 95% coverage interval derived from the simulation is narrower and more precise than the one obtained from the GUM approach, yielding an expanded uncertainty of ±0.62% compared to ±0.69%. This demonstrates the MCS’s ability to provide a more refined uncertainty estimate for this model.

## 4. Discussion

The MCS was performed with 1,000,000 iterations to achieve statistically stable results, following the recommendations in GUM Supplement 1. This computational approach generated reliable estimates of both the measured content value and its associated standard uncertainty.

The simulation outputs presented in Table 5 reveal several important findings. The 95% confidence interval obtained through MCS showed two notable characteristics compared to conventional GUM analysis: it was slightly asymmetric and approximately 19.4% more precise than the interval produced by the GUM method.

Beyond the theoretical comparison, the practical strengths of MCS are important for modern pharmaceutical analysis. Unlike GUM’s bottom-up approach, which relies on linear approximations and can become complex when modeling correlations or nonlinear systems, MCS offers a more flexible framework that can readily incorporate complex model structures. This is especially advantageous for methods involving nonlinear calibration curves, such as those encountered in LC-MS or immunoassays. Furthermore, MCS provides a direct numerical estimation of the output distribution, enabling the accurate determination of confidence intervals without relying on the central limit theorem or assumptions of normality. This capability supports risk-based quality decision-making by allowing a more realistic assessment of measurement reliability, particularly when results approach specification limits.

While the current study focused on a straightforward HPLC-UV assay where both methods showed agreement, the MCS approach demonstrates practical advantages for more complex analytical systems where the GUM’s assumptions may not hold.

The findings demonstrate that MCS provides a more rigorous approach to uncertainty estimation, particularly when dealing with mixed input distributions. The key advantage is that it calculates coverage factors directly from the simulated data distribution (yielding *k* = 1.9529) rather than assuming a standard value (*k* = 2), and it more accurately models the combined effects of all input uncertainties without relying on linearizing approximations. This leads to a more precise confidence interval and a more accurate representation of the output distribution shape.

In our study, the output distribution for MPZ content obtained via MCS (Figure 6) was effectively symmetric and approximately normal.

Figure 6 distribution of the Metopimazine (MPZ) content (%) obtained from the Monte Carlo Simulation (*M* = 10^6^ trials). The histogram shows the frequency of the simulated results. The symmetric, bell-shaped profile visually confirms that the output is approximately normally distributed, which is supported by the low calculated skewness value of 0.0085.

Table 6 shows strong agreement between MCS and GUM results, with minor differences (0.028% in mean values, 6.9% in uncertainty estimates) within acceptable limits for pharmaceutical quality control. The close correspondence between these two fundamentally different calculation methods can be primarily attributed to the linear nature of the mathematical models used in this analysis.

This agreement serves as an important validation of both methodologies. The MCS confirms the reliability of the simpler GUM approach for this application, while offering distinct advantages for more complex, nonlinear systems where analytical solutions may be inadequate.

Beyond the methodological comparison, the rigorous uncertainty framework established here has direct and significant implications for pharmaceutical quality control and regulatory compliance. Firstly, the detailed uncertainty budget (Table 3) provides a clear roadmap for laboratories to meet the requirement for measurement uncertainty estimation under ISO/IEC 17025 audits [10]. By identifying sample volume and calibration as the dominant sources of uncertainty (Figure 4), our study offers laboratories actionable insights on where to focus their method optimization and equipment qualification efforts to maximize data quality. Secondly, the determination of an expanded uncertainty (U = 0.69%) that occupies only a small fraction (6.9%) of the acceptance limit (±10%) provides strong objective evidence that the method is ‘fit-for-purpose’ and suitable for its intended use of batch release. This quantitative demonstration is far more powerful than a simple statement of compliance. Finally, this uncertainty value is critical for making informed conformity decisions [6]. When a measured result is close to the specification limit, the known uncertainty can be used to calculate the risk of a false acceptance or false rejection decision, moving from pass/fail testing towards a more sophisticated, risk-based quality assurance paradigm as encouraged by modern regulatory guidelines.

The strong agreement between the GUM and MCS results, with both $d_{low}$ (0.0376) and $d_{high}$ (0.0474) falling well below the validation threshold *δ* (0.05) (Table 7), was expected. This convergence occurs because the measurement model is effectively linear and the output quantity is normally distributed within the working range of this assay, which are the key assumptions underlying the GUM framework.

While our current analysis used the appropriate *δ* = 0.05 corresponding to *u*(*y*) ≈ 0.3, we note that a stricter tolerance (e.g., *δ* = 0.005) would require an order-of-magnitude smaller standard uncertainty (*u*(*y*) ≈ 0.03). Such artificial tightening of δ would invalidate the GUM approach in this case, as *d_high_* (0.047) > 0.005. This demonstrates how proper δ selection must be based on the actual measurement uncertainty magnitude.

For linear models with normally distributed measurands, the ISO-GUM approach remains practical and widely applicable. It offers a recognized methodology that does not require specialized software. However, its limitations become apparent with complex systems, particularly in calculating sensitivity coefficients and requiring assumptions about input distributions and Gaussian outputs. In contrast, MCS demonstrates superior capability for handling nonlinear models without simplification, generating reliable coverage intervals without distributional assumptions, and propagating complete distribution information. This distinction proves particularly relevant for modern pharmaceutical analysis using techniques like HPLC-UV, GC-MS, and spectrophotometry. While our method agrees with simple, linear systems—validating GUM’s utility for routine analyses—MCS becomes essential when facing model nonlinearities or when distribution assumptions cannot be verified. The MCS approach offers advantages by eliminating Taylor series approximation errors and providing empirical output distributions. This makes it valuable for obtaining precise uncertainty estimates without relying on Gaussian assumptions. These findings provide analytical scientists with a critical framework for selecting appropriate uncertainty estimation methods based on the complexity of the measurement scenario, rather than relying solely on conventional practice.

## 5. Conclusions

This study provides an evaluation of uncertainty estimation methods for the HPLC-UV determination of Metopimazine, comparing the conventional GUM approach with MCS. Our findings demonstrate that both methods yield consistent results for this application, with the GUM method being sufficient for routine quality control due to the linearity of the analytical response and normally distributed residuals. The MCS approach, while more computationally intensive, offers advantages in detecting subtle distributional characteristics and providing more precise coverage intervals. For the Metopimazine HPLC-UV assay specifically, the differences between methods were minimal, supporting the continued use of the GUM framework for standard pharmaceutical analysis. The methodologies and the comparative framework developed here, particularly the identification of sample volume and calibration as the dominant uncertainty sources, are relevant for the quality control of Metopimazine. Given the structural and methodological similarities within the phenothiazine class, this work could provide a useful starting point for uncertainty analysis of other related pharmaceuticals; however, this would require future, compound-specific validation. Ultimately, this study establishes a robust, validated model for uncertainty analysis in drug substance quantification that aligns laboratory practices with international standards and strengthens confidence in the quality of critical antiemetic medication.

## Figures and Tables

**Table 1:** Uncertainty Budget for Volumetric Glassware Used in MPZ Dilution. The squared relative uncertainties are used in the propagation of uncertainty to compute the combined standard uncertainty.

Instrument	Volume (mL)	Calibration	Standard Uncertainty of Calibration	Temperature Effect	Standard Uncertainty of Temperature	Uncertainty of Volume	Relative Uncertainty of Volume	(Relative Uncertainty)^2^	Relative Uncertainty of Dilution
Pipette	2	0.01	0.004082	0.00168	0.00097	0.0042	0.0021	0.000004	0.002683
Pipette	5	0.015	0.00612	0.0042	0.00242	0.00659	0.0013	0.000002	
Flask	25	0.04	0.01633	0.021	0.01212	0.02034	0.0008	0.000001	
Flask	100	0.1	0.04082	0.084	0.0485	0.06339	0.0006	0.00000	

**Table 2:** System Suitability Test Results and Associated Standard Uncertainty for HPLC-UV Parameters.

	Mean	Acceptance Criterion	CV (%)	Standard Deviation	Standard Deviation of the Mean	*U*(*_HPLC-UV_*)
Retention time	3.808	% CV < 1.0%	0.08	0.003807	0.0015542	0.0022
Area	70.509	% CV < 2.0%	0.1	0.001568	0.00064013	

**Table 3:** Uncertainty Budget for Metopimazine HPLC-UV Assay.

Symbol	Description	Value *X*	Standard Uncertainty *u*(*X*)	Relative Standard Uncertainty *u*(*X*)/*X*
*δr*	Repeatability	1	0.0005	0.0005
*C_x_*	Predicted Concentration by calibration	1	0.0004	0.002
*A_t_*	The peak area of Metopimazine in the test solution	70.509	0.0022	3.12017 × 10^−5^
*A_st_*	The peak area of Metopimazine in the standard solution.	70.714	0.0022	3.11112 × 10^−5^
*P_t_*	weight of reference substance in mg	100	0.01155	0.0001155
*V_t1_*	The volume of the standard stock solution in mL	100	0.06339	0.0006339
*V_t2_*	dilution volume of volume taken from the stock solution in mL	25	0.02034	0.0008136
*V_p_*	The volume taken from the standard stock solution in mL	5	0.00659	0.001318
*V_t_*	The volume of the test solution	100	0.06339	0.0006339
*V_Sample_*	Volume of specimen in mL	2	0.00420	0.0021
*P*	Purity of Metopimazine in %	0.9999	0.000058	5.80058 × 10^−5^

**Table 4:** Probability Distribution Functions (PDFs) Assigned to Input Parameters for the HPLC-UV Quantification of Metopimazine (MPZ).

Parameters	Description	Type of Distribution	Mean	Standard Deviation	*a*	*b*
*δ_r_*	Repeatability	Normal	1	0.0005	*	*
*C_x_*	Predicted Concentration by calibration	Normal	0.2	0.0004	*	*
*A_t_*	The peak area of Metopimazine in the test solution	Normal	70.509	0.0022	*	*
*A_st_*	The peak area of Metopimazine in the standard solution.	Normal	70.714	0.0022	*	*
*V_Sample_* (*C*)	Volume of specimen in mL	Triangular	2	*	1.99	2.01
*V_Sample_* (*T*)		Rectangular	2	*	1.99832	2.00168
*P_t_*	weight of reference substance in mg	Rectangular	100	*	99.98	100.02
*V_t1_* (*C*)	Dilution volume of standard stock solution in mL (100 mL)	Triangular	100	*	99.9	100.1
*V_t1_* (*T*)		Rectangular	100	*	99.916	100.084
*V_t2_* (*C*)	Dilution volume of volume taken from the stock solution in mL (25 mL)	Triangular	25	*	24.96	25.04
*V_t2_* (*T*)		Rectangular	25	*	24.979	25.021
*V_p_* (*C*)	Volume taken from the standard stock solution. in mL (5 mL)	Triangular	5	*	4.985	5.015
*V_p_* (*T*)		Rectangular	5	*	4.9958	5.0042
*V_t_* (*C*)	Volume of the test solution (100 mL)	Triangular	100	*	99.9	100.1
*V_t_* (*T*)		Rectangular	100	*	99.916	100.084
*P*	Purity of Metopimazine in %.	Rectangular	0.9999	*	0.99984	0.999958

Note: The asterisk (*) indicates that the value is not applicable or not defined for the given distribution type. Legend: (*C*) represents the Calibration tolerance of the glassware, which we model with a triangular distribution. (*T*) represents the Temperature effect, which we model with a rectangular distribution. The repeatability component for volumetric operations is included in the overall method precision term (*u*(*δr*)) to avoid double-counting.

**Table 5:** Statistical Output from MCS Uncertainty Analysis for the HPLC-UV Quantification of MPZ.

Parameters	Estimated Values
Mean (content %)	99.3822
Median (%)	99.3817
Standard uncertainty %	0.3189
Expanded uncertainty	0.6228
Skewness	0.0085
Coverage factor *k*	1.9529
Confidence interval for 95%	[98.7623; 100.0079]

**Table 6:** Uncertainty Analysis Results—GUM versus MCS Approaches.

		MPZ Assay	
		GUM	MCS
Mean value		99.41	99.3822
Standard uncertainty		0.3426	0.3189
Coverage factor		2	1.9529
Expanded uncertainty		0.6853	0.6228
Coverage interval of 95%	Lower limit	98.4747	98.7623
	Upper limit	100.0953	100.0679

**Table 7:** Comparative Validation of Measurement Uncertainty: GUM vs. MCS for MPZ Assay.

MPZ	**GUM**		**MCS**		***D_low_***	***D_high_***	***δ***
	*Y*	99.41	*Y_Low_*	98.7623	0.0376	0.0474	0.05
	*U*	0.6853	*Y_high_*	100.0679			

## Data Availability

The original contributions presented in this study are included in the article/Supplementary Materials. Further inquiries can be directed to the corresponding author.

## Supplementary Materials

The following supporting information can be downloaded at: https://www.mdpi.com/article/10.3390/pharmaceutics17101316/s1.
