# Supplementary material for: Metrological Evaluation of Metopimazine HPLC Assay: ISO-GUM and Monte Carlo Simulation Approaches
Source: Pharmaceutics. 2025 Oct 10;17(10):1316. doi: 10.3390/pharmaceutics17101316 (PMC12567434; doi:10.3390/pharmaceutics17101316)
Supplement: Supplementary file 1 [file pharmaceutics-17-01316-s001.zip › pharmaceutics-3879435-supplementary.pdf]

Supplementary material

Metrological Evaluation of Metopimazine HPLC Assay: ISO-GUM and Monte Carlo Simulation Approaches

Hasnaa Haidara [1,2,*], Eman A. Assirey [3], Taoufiq Saffaj [2] and Bouchaib Ihssane [4]

[1] Laboratory of Organic Chemistry, Catalysis, and Environment, Faculty of Sciences, Ibn Tofail University, 133, Kenitra 14000, Morocco
[2] Laboratory of Applied Organic Chemistry, Faculty of Sciences and Technology, Fez-Sidi Mohamed Ben Abdellah University, Immouzer Road, Fez 2202, Morocco; taoufiq.saffaj@usmba.ac.ma
[3] Department of Chemistry, College of Science, Taibah University, Al-Madinah Al-Munawarah 41411, Saudi Arabia; eassirey@taibahu.edu.sa
[4] Laboratory of Inorganic and Organic Materials (LPCMIO), Materials Science Center (MSC), School Normal Superior Physio-Chemical, University Mohammed V, ENS Avenue Mohamed Bel Hassan El Ouazzani,
Takaddoum, Rabat 5118, Morocco; chouaibihssane@yahoo.fr
* Correspondence: hasnaa.haidara@usmba.ac.ma

***Table S1**: Statistical parameters of the calibration curve.*

Parameter	*Value*	*Standard Error*	*95% Confidence Interval*
Slope (a_1)	*332.14 AU·mL/mg*	*2.29*	*327.19 to 337.09*
Intercept (a_0)	*3.75 AU*	*0.48*	*2.71 to 4.79*
Residual Std. Dev. (S_{res})	*0.27 AU*		
R-squared (R^2)	*0.999*		
SSD_x	*0.048 $(mg/mL)^2$*		

**AU: Arbitrary Units. CI calculated with t(0.05, 13) = 2.16.*

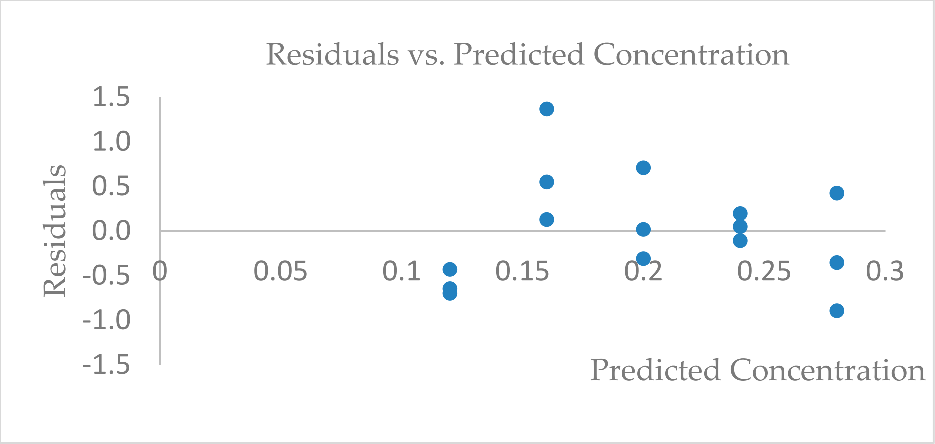

Figure S1: *Plot of residuals versus predicted concentration for the Metopimazine calibration curve (n=15).*

- *The random scatter confirms the homoscedasticity assumption required for the unweighted regression model in Eq. (15).*

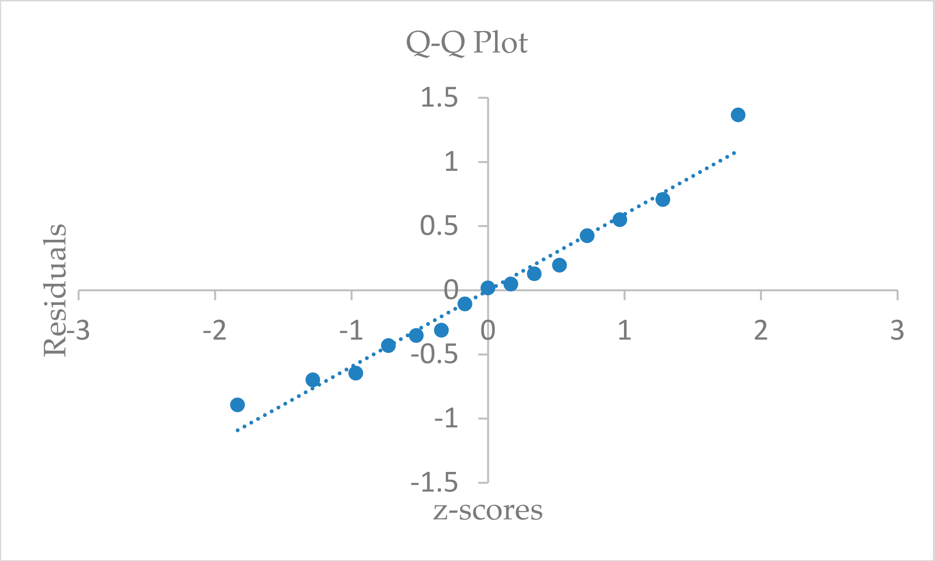

Figure S2: *Normal quantile-quantile (Q-Q) plot of residuals from the Metopimazine calibration curve.*

- *The close alignment of the points with the reference line indicates normality of the residuals, validating the statistical approach for uncertainty estimation.*
